# A quick protocol for the preparation of mouse retinal cryosections for immunohistochemistry

**DOI:** 10.1098/rsob.210076

**Published:** 2021-07-28

**Authors:** Jialiang Yang, Tongdan Zou, Fang Yang, Zilong Zhang, Chen Sun, Zhenglin Yang, Houbin Zhang

**Affiliations:** ^1^ The Key Laboratory for Human Disease Gene Study of Sichuan Province and Institute of Laboratory Medicine, Sichuan Provincial People's Hospital, University of Electronic Science and Technology of China, Chengdu, Sichuan, People's Republic of China; ^2^ State Key Laboratory of Southwestern Chinese Medicine Resources, Pharmacy School, Chengdu University of Traditional Chinese Medicine, Chengdu, Sichuan, People's Republic of China; ^3^ Institute of Chengdu Biology, Sichuan Translational Medicine Hospital, Chinese Academy of Sciences, Chengdu, Sichuan, People's Republic of China; ^4^ Research Unit for Blindness Prevention of Chinese Academy of Medical Sciences (2019RU026), Sichuan Academy of Medical Sciences and Sichuan Provincial People's Hospital, Chengdu, Sichuan, People's Republic of China

**Keywords:** mouse retina, cryosection, immunohistochemistry, photoreceptors, Super Glue

## Abstract

Immunohistochemistry (IHC) using mouse retinal cryosections is widely used to study the expression and intracellular localization of proteins in mouse retinas. Conventionally, the preparation of retinal cryosections from mice involves tissue fixation, cryoprotection, the removal of the cornea and lens, embedding and sectioning. The procedure takes 1–2 days to complete. Recently, we developed a new technique for the preparation of murine retinal cryosections by coating the sclera with a layer of Super Glue. This enables us to remove the cornea and extract the lens from the unfixed murine eye without causing the eyecup to collapse. In the present study, based on this new technique, we move a step forward to modify the conventional protocol. Unlike in the conventional protocol, in this method, we first coat the unfixed mouse eyeball on the sclera with Super Glue and then remove the cornea and lens. The eyecup is then fixed, cryoprotected and sectioned. This new protocol for the preparation of retinal cryosections reduces the time for the procedure to as little as 2 h. Importantly, the new protocol consistently improves the morphology of retinal sections as well as the image quality of IHC. Thus, this new quick protocol will be greatly beneficial to the community of visual sciences by expediting research progress and improving the results of IHC.

## Graphical abstract

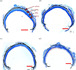

## 1. Introduction

Retinal degeneration is one of the leading causes of irreversible blindness involving the death of photoreceptors. Photoreceptors are specialized, light-sensitive neuronal cells. Structurally, a photoreceptor cell comprises several morphologically distinct compartments, including the outer segment, connecting cilium, inner segment, nucleus and synaptic terminal. The unique topology of photoreceptors and their inability to divide prevent the establishment of a photoreceptor cell line.

Recently, retinal organoids derived from human-induced pluripotent stem cells (iPSCs) have attracted significant attention as model systems to investigate the aetiology of retinal diseases and develop potential therapeutic strategies [1]. Despite the progress made on organoid culture, animal models remain the most popular system for studies on the mechanisms of human diseases, including retinal diseases. Among all animal models, mouse models are the most widely used and provide tremendous insight into the functional and disease mechanisms of retinas [2]. In the past decades, numerous murine models have been developed to mimic human retinal diseases by employing genetic modification [3,4], physical injury [5] or intraocular virus injection [6]. Additionally, dozens of mouse strains carrying spontaneous mutations have been identified [2,7,8].

To understand the function of genes expressed in the mouse retina or reveal the molecular events underlying retinal diseases, it is important to detect the expression and localization of proteins in the retina by using immunohistochemistry (IHC). IHC using cryosections prepared from fixed retinas has become a routine technique due to its convenience and good preservation of the immunoreactivity of antigens in the retina. The mouse retina, however, is extremely fragile and susceptible to detachment from the eyecup caused by the mechanical forces during tissue processing. Breakage in the outer segment of the photoreceptors or the connecting cilium is also commonly found in retinal cryosections. To tackle these problems, we have recently developed a novel method to process mouse eyecups by coating the sclera with a layer of Super Glue. This prevents the deformation of the eyecup, which markedly improves the quality of immunostaining results [9]. We designate this method as ‘the old-glue method’.

To produce retinal cryosections, the eyeballs must be fixed and cryoprotected before embedding [10,11]. Traditionally, we fixed mouse eyeballs for 2 h to obtain retinal sections with good morphologies [3,11]. Although a shorter fixation time is used by some researchers [10], the morphology of photoreceptors in the sections is not as good. Cryoprotection in the sucrose solution may take up to 12 h. This is because the retina enclosed within the sclera is relatively rigid, and it is a relatively slow process for the fixative and sucrose solution to access the retina. On the other hand, prior to fixation, the sclera is relatively soft. It is difficult to remove the cornea and lens and expose the retina to allow it better access to the fixative, because without the cornea and lens, the eyecup would collapse before fixation. Altogether, this process usually lasts over a day. The lengthy process may slow down the progress of experiments, which, in some cases, must obtain results quickly. However, this difficulty can be overcome by employing the old-glue method we recently developed.

In the present study, inspired by the method for the preparation of retinal cryosections from unfixed eyeballs [9], the conventional protocol of tissue fixation and cryoprotection is further modified, which greatly shortens the time required for tissue fixation and cryoprotection from 2 h and overnight to 30 min and 30 min, respectively. This reduces the time from 2–3 days to 1–2 days for the entire IHC procedure, including tissue processing, tissue sectioning and immunostaining. Moreover, this quick protocol (designated as ‘the new method’ or ‘the new protocol’ here) does not expedite the retinal IHC process at the expense of quality. Photoreceptor morphology and the immunofluorescent images produced by this new protocol are comparable to or better than those from the traditional one.

## 2. Results

### 2.1. The quick protocol improves the morphology of retinal sections

**Figure 1.**
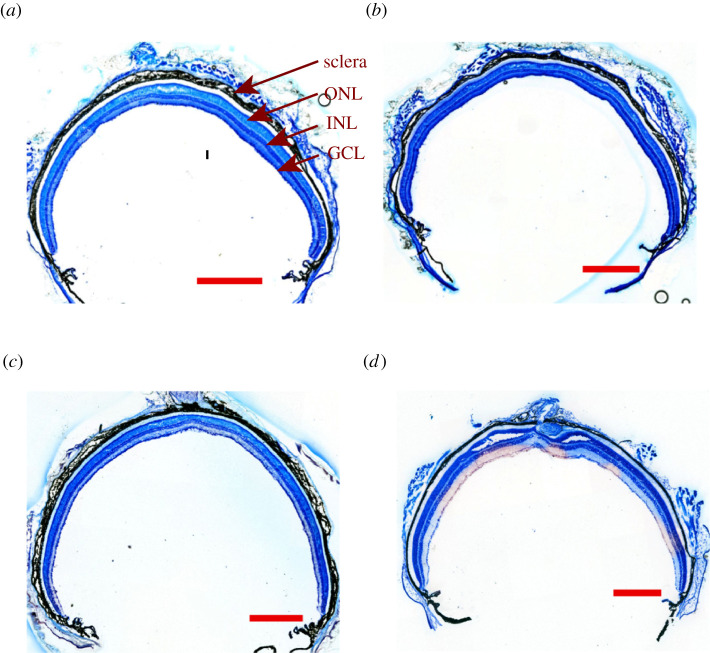
Morphological study of retinal cryosections. The retinal sections from a one-month-old mouse were stained with Toluidine blue. The retinal sections produced from mouse eyecups were fixed for 10 min (*a*), 20 min (*b*) and 30 min (*c*) using the new method. (*d*) The retinal section produced by the conventional method. Scale bar, 500 µm. ONL, outer nuclear layer; INL, inner nuclear layer; GCL, ganglion cell layer.

The traditional protocol for the preparation of retinal cryosections requires the fixation of eyeballs for 2 h prior to embedding. To reduce the time required to fixate the mouse's eye, the sclera of the unfixed eyeball was coated with a layer of Super Glue, and the cornea and lens were removed. The absence of the cornea and lens facilitated the fixative to infiltrate the tissue within the eyecup, which could speed up the tissue fixation. This also shortened the time required for cryoprotection in the sucrose solution. We varied the fixation time and cryoprotection time and found that 10, 20 or 30 min of fixation combined with 30 min of cryoprotection was sufficient to produce cryosections with great morphologies (all animals used in this study were C57Bl6/J unless specified) (figure 1*a*–*c*). The fixation time had little effect on the gross of the retinal sections. No retinal detachment was found throughout the retinal section prepared by this new method, although tiny holes or a very small area of tissue breakage could occasionally be found in the outer nuclear layer (figure 1*a*). By comparison, the retinal sections prepared by the conventional method had a large area of broken tissues in the central part of the section (figure 1*d*). The new protocol was tested on mice of different ages ranging from P12 to 1-year old, and all yielded similar results. Therefore, the new protocol not only shortens the time required for preparing retinal cryosections but also greatly improves the overall morphology of the retinal section.

### 2.2. The quick protocol improves the photoreceptor morphology in IHC

**Figure 2.**
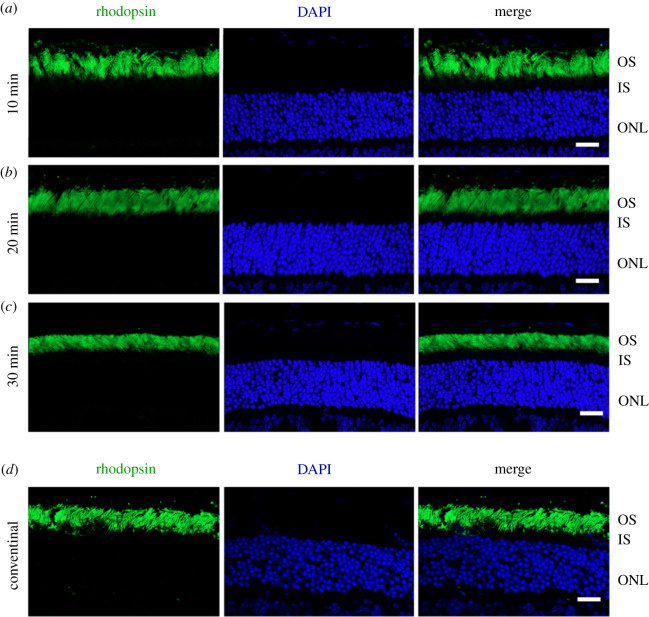
Immunohistochemical study of retinal sections to label rod photoreceptors. (*a*–*c*) Representative retinal sections produced using the new method from the eyecup fixed for 10 min (*a*), 20 min (*b*) and 30 min (*c*). (*d*) Representative retinal sections produced by the conventional method. Sections were immunostained with anti-rhodopsin (green) and counterstained with DAPI (blue). Scale bar, 20 µm. OS: outer segment; IS: inner segment; ONL: outer nuclear layer.

**Figure 3.**
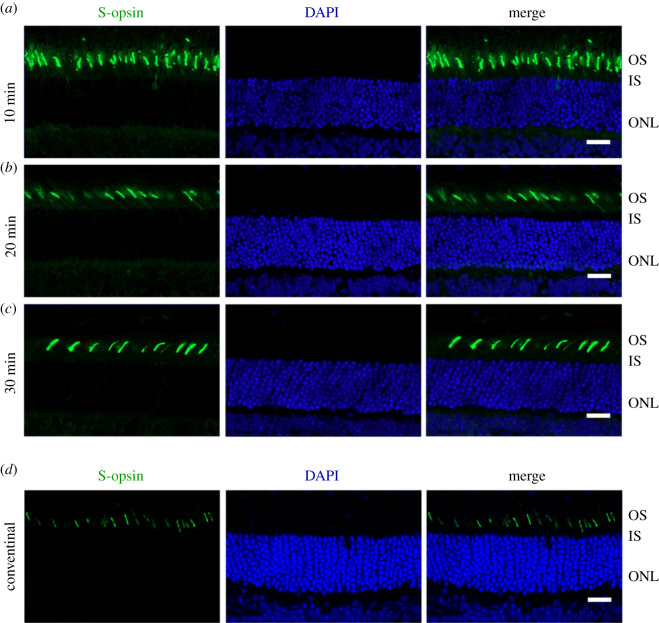
Immunohistochemical study of retinal sections to label cone photoreceptors. (*a*–*c*) Representative retinal sections produced using the new method from the eyecup fixed for 10 min (*a*), 20 min (*b*) and 30 min (*c*). (*d*) Representative retinal sections produced by the conventional method. Sections were immunostained with anti-S-opsin (green) and counterstained with DAPI (blue). Scale bar, 20 µm. OS: outer segment; IS: inner segment; ONL: outer nuclear layer.

**Figure 4.**
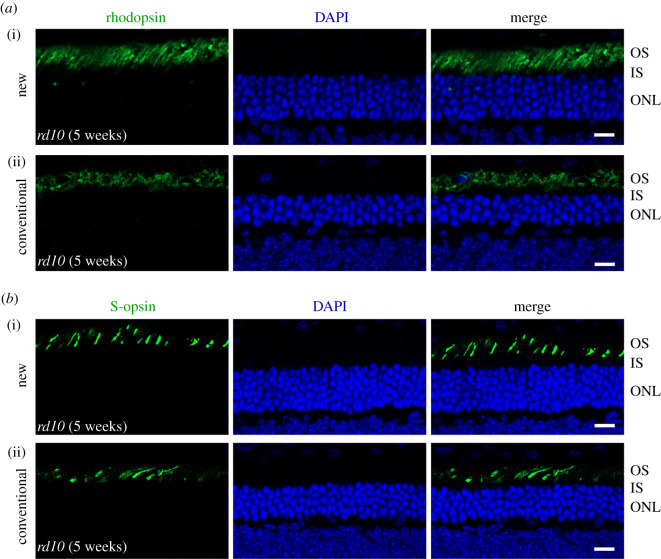
Immunohistochemical study of *rd10* retinal sections. (*a*) Representative retinal sections produced from five-week-old *rd10* mice by the new protocol (i) and conventional method (ii), respectively. The sections were immunostained with anti-rhodopsin (green) and counterstained with DAPI (blue). (*b*) Representative retinal sections produced from five-week-old *rd10* mice by the new protocol (i) and conventional method (ii), respectively. The sections were immunostained with anti-S-opsin (green) and counterstained with DAPI (blue). Scale bar, 20 µm. OS: outer segment; IS: inner segment; ONL: outer nuclear layer.

To test whether the new protocol affects the photoreceptor morphology or the immunoreactivity of proteins in retinal sections, the retinal sections were immunostained to label rhodopsin, a rod outer segment-specific marker. The immunostaining results of cryosections by the new protocol were compared from retinas fixed for 10, 20 and 30 min. As shown in figure 2*a*, the rhodopsin antibody specifically and intensely stained the photoreceptor outer segments, indicating that the new protocol or the length of fixation did not affect the immunoreactivity of rhodopsin in the outer segments. While all sections yielded acceptable results, the quality of the staining result increased when the fixation time extended from 10 min to 30 min. The best result was achieved from the retina fixed for 30 min, as it showed more intact photoreceptors and better-aligned photoreceptor outer segments. By contrast, the photoreceptor outer segments were less organized in the retinal sections produced by the conventional method. Occasional broken outer segments and holes were found in the outer segment layer (figure 2*b*). However, the fluorescent signals were more solid in the outer segments, reflecting better fixation. Thus, when the fixation time in the new method reaches 30 min, the overall quality of immunostaining results for rods is far better, compared to the old method. We also immunostained the sections using an antibody against S-opsin, an S-cone outer segment-specific marker. As shown in figure 3*a*, regardless of fixation time, the morphologies of the cone outer segments in sections produced by the new method were indistinguishable, indicating that 10 min of fixation is sufficient to produce high-quality immunofluorescent images of cones The quality of the cone images by the new method was slightly better than that by the conventional method (figure 3*a*,*b*). Additionally, we tested the new method on the *rd10* mice whose retinas start to degenerate within the first month after birth. The rod photoreceptor morphology appeared better in the section prepared by the new method than by the conventional method (figure 4*a*). The cone photoreceptor morphology was also preserved better in the section prepared by the new method (figure 4*b*). Therefore, while the new method does not affect the immunoreactivity of antigens in retinal sections, it improves the quality of immunostaining results for healthy and degenerative retinas, especially for rod photoreceptors.

## 3. Discussion

In the conventional protocol, it is impossible to remove the cornea and lens before the eyeball is fixed well. If it is not fixed well, the eyecup will collapse after the cornea and lens are removed, which could cause severe damage to the photoreceptor morphology in the section. Previously, we developed the old-glue method that allows the cornea and lens to be removed from the unfixed eyeball without causing deformation by coating the eyeball with a layer of Super Glue [9]. The study described here further modifies that method and greatly reduces the time required for the production of retinal cryosections.

The retina is a very fragile tissue. In the conventional protocol for preparing retinal sections, multiple actions could cause retinal detachment or the breakage of photoreceptors in the outer segment or connecting cilium. In the conventional protocol, an incision must be made on the cornea before the eyeball is fixed. The incision allows the fixative to infiltrate the inner space of the eyeball. More importantly, the incision eliminates the pressure and osmotic difference between the outside and inside of the eyeball, thereby preventing shrinkage of the eyeball during fixation. This shrinking could cause severe retinal detachment and deteriorate the gross morphology of the resultant retinal sections. To make an incision on the cornea, however, the cornea must be pinched by tweezers, which facilitates cutting by scissors or poking by a needle. Pinching the cornea could also introduce retinal detachment. In the new method described in this study, a pipette tip is attached to the cornea with Super Glue. The pipette tip serves as a ‘handle’. By holding this handle, it is easy to manoeuvre the position and orientation of the eyeball. The cornea can be cut away without it being pinched, avoiding any deformation of the eyeball and minimizing retinal detachment.

Our previous study indicates that coating the sclera with a layer of Super Glue can prevent any distortion of the eyeball during processing and improve the morphology of retinal sections [9]. In that study, Super Glue was applied to the surface of the sclera with a pipette, and then Super Glue was spread on the sclera. However, spreading Super Glue evenly on the sclera requires some practice. Additionally, an excess amount of Super Glue may spread to the cornea, which could make the eyeball stick to the supporting surface. In the new method, the eyeball is dipped into the Super Glue solution by holding the pipette tip handle. It requires no spreading, and Super Glue can be coated more evenly. Thus, the coating can be completed within 1–2 s with little effort or practice. One of the characteristics of Super Glue is that it hardens instantly after it encounters water. Taking advantage of this property, the eyeball is dipped into the PBS saline immediately after the eyeball is removed from the Super Glue solution. This way, Super Glue is more evenly coated on the sclera and takes less time to harden. This new process further improves the morphology of retinal sections prepared from the old-glue method.

Like the old-glue method [9], coating the sclera with Super Glue using this new protocol also preserves the morphology of photoreceptors very well. The direct exposure of the retina to the fixative allows the retina to be fixed within a shorter time frame. This substantially cuts the time required for fixation and cryoprotection. Perfusion of the mice using paraformaldehyde (PFA) could also shorten the time for tissue fixation. However, perfusion uses a large amount of PFA, which is harmful for operators and must be handled in a hood with good ventilation.

Another operation in the conventional protocol that could cause retinal detachment is the embedding process. During embedding, the eyecup is dragged and rolled to adjust its position and orientation in the sticky OCT medium. Although operated gently, this dragging and rolling could cause retinal detachment and the breakage of photoreceptor cells as well. The coating on the sclera could avoid this problem. The new glue method coats the sclera with Super Glue from the very beginning of the procedure, providing better protection for the tissue compared to the old-glue method.

In summary, based on the old-glue method, we further modified the protocol for producing retinal cryosections from mouse eyes. This new method cuts the time for the procedure of retinal section production and yields a great result at IHC. This new method expedites research involving retinal IHC and improves the quality of IHC results.

## 4. Material and methods

### 4.1. Animals

The wild-type C57Bl6/J mouse strain and *rd10* mouse strain (obtained from The Jackson Laboratories, USA) were used in all experiments in this study. The mice were housed in the animal facility at Sichuan Provincial People's Hospital. All procedures were approved by the Animal Care and Use Committee of the Sichuan Provincial People's Hospital and in accordance with the guidelines of the U.S. National Institutes of Health Guide for Care and Use of Laboratory Animals.

### 4.2. Preparation of retinal cryosections using the new method

**Figure 5.**
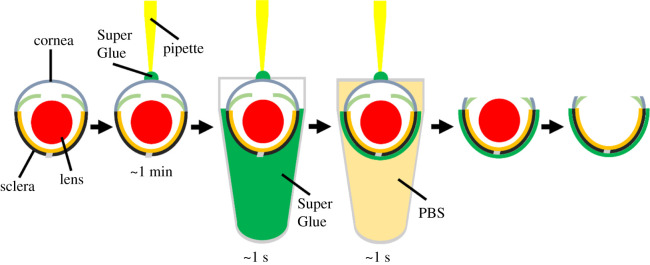
Schematic showing the processing of mouse eyeballs using the new method. An unfixed, fresh mouse eyeball is placed under a dissecting microscope with the eyecup facing up. A pipette with Super Glue at the tip contacts the central part of the cornea. After about 1 min, the Super Glue hardens, and the eyeball and pipette are glued together. By holding the pipette, the eyeball is dipped briefly into Super Glue contained in a microcentrifuge tube, leaving the cornea above the liquid surface. Subsequently, the eyeball coated with Super Glue is submerged into 1XPBS. In PBS, the Super Glue hardens instantly. Then, the cornea is removed and the lens is extracted.

The procedure for preparing retinal cryosections was modified from the previous report [2], as illustrated in figure 5. The mouse aged one month was euthanized by cervical dislocation. The eyeballs were enucleated from the mouse and rinsed briefly with 1XPBS (phosphate-buffered saline, pH 7.4). The temporal side of the eye was marked by a blue Sharpie pen. The eyeball was placed on a Petri dish with the eyecup facing up. Excess PBS was removed by Kimwipe paper, with the surface of the eyeball remaining moist. A 10 µl pipette (or a better result can be achieved by using a segment of 17-gauge nylon tennis string, about 3 cm long) was dipped briefly into Super Glue (also named Krazy Glue or Glue 502, purchased from convenient stores) contained in a mini-centrifuge tube. It was removed immediately after, and the tip of the pipette made contact with the central cornea. After roughly 1 min, the residue glue left on the tip of the pipette hardened and bonded the pipette and eyeball together. The eyeball was dipped briefly into the Super Glue in the mini-centrifuge tube by holding the pipette (only the sclera part was immersed in the Super Glue) and was removed quickly. The eyeball was completely immersed in 1XPBS saline for about 1–2 s and then removed. The Super Glue hardened immediately in PBS. Excess PBS on the eyeball was removed by Kimwipe paper. Under a dissecting microscope, a syringe needle was used to poke a hole at the edge of the cornea. The cornea (attached to the pipette) and lens were carefully removed. The eyecup was submerged in 4% PFA in PBS and fixed at room temperature for 30 min. Subsequently, the eyecup was transferred to 30% sucrose in PBS for cryoprotection at room temperature for 30 min. It was then embedded into the optimal cutting temperature (OCT) compound, with the sagittal plane of the eye parallel to the bottom of the cryomold. After it was frozen in a −80°C freezer, the tissue was sectioned on a cryostat at 12 µm.

### 4.3. Preparation for retinal cryosections using the conventional method

The eyeball enucleated from the euthanized mouse as described above was immersed in 4% PFA in PBS for fixation on ice for 10 min. The eyeball was removed from the fixative, and an incision was made on the cornea. It was returned to 4% PFA and fixed for an additional 2 h on ice. The eyeball was then transferred to 30% sucrose in PBS for cryoprotection at 4°C overnight. After the cornea was removed and the lens was extracted, the eyecup was embedded and sectioned as described above.

### 4.4. Immunostaining of retinal sections

The retinal sections were permeabilized using 5% donkey serum in PBS containing 0.2% Triton X-100, as described previously. The sections were incubated with primary polyclonal anti-rhodopsin antibody (1 : 900, VPP, custom made [12]) and polyclonal anti-S-opsin antibody (1 : 500, Millipore, USA) at 4°C overnight. Alexa488-conjugated goat anti-rabbit secondary antibody (ThermoFisher Scientific, USA) was diluted at 1 : 300 and incubated at room temperature for 1 h. The sections were mounted with an anti-fade mounting medium (Sigma, USA). All the antibodies were diluted in 5% donkey serum prepared in 1XPBS supplemented with 0.2% Triton X-100. The images were acquired by a Zeiss LSCM 900 confocal microscope.

### 4.5. Toluidine blue staining

The sections were rinsed with 1XPBS for 2 × 15 min to remove the embedding medium. A drop of 0.025% Toluidine blue (Sigma, USA) in 1% sodium borate was applied to the sections and allowed for staining for 30 s. Washing with tap water followed. Images of the sections were taken with a Zeiss light microscope.

## Data accessibility

This article does not contain any additional data.
